# Crystal structure of 16-hy­droxy-4,4,10,13,14-penta­methyl-17-(6-methyl­hept-5-en-2-yl)-4,5,6,9,10,11,12,13,14,15,16,17-dodeca­hydro-1*H*-cyclo­penta­[*a*]phenanthren-3(2*H*)-one

**DOI:** 10.1107/S2056989015010592

**Published:** 2015-06-13

**Authors:** Jun-Jun Ge, Pian Chen, Xiao-Xia Ye

**Affiliations:** a School of Pharmacy, Wenzhou Medical University, Wenzhou 325035, People’s Republic of China

**Keywords:** crystal structure, triterpenoid, *Melia azedarac*h, O—H⋯O hydrogen bonds

## Abstract

The title compound, C_30_H_48_O_2_, contains a fused four-ring triterpenoid system. In the mol­ecule, the two cyclo­hexane rings adopt a chair conformation and a twist boat conformation, respectively, the central cyclo­hexene ring adopts a half-chair conformation whereas the five membered ring adopts an envelope conformation. In the crystal, O—H⋯O hydrogen bonds between the hy­droxy and carbonyl groups of adjacent mol­ecules link the mol­ecules into supra­molecular chains propagating along the *b*-axis direction.

## Related literature   

For biological applications of triterpenoid compounds, see: Faizi *et al.* (2002 ▸); Wang *et al.* (2011 ▸); Dong *et al.* (2012 ▸). For isolation of the title compound from the barks of *Melia azedarach*, see: Chang & Chiang (1969 ▸).
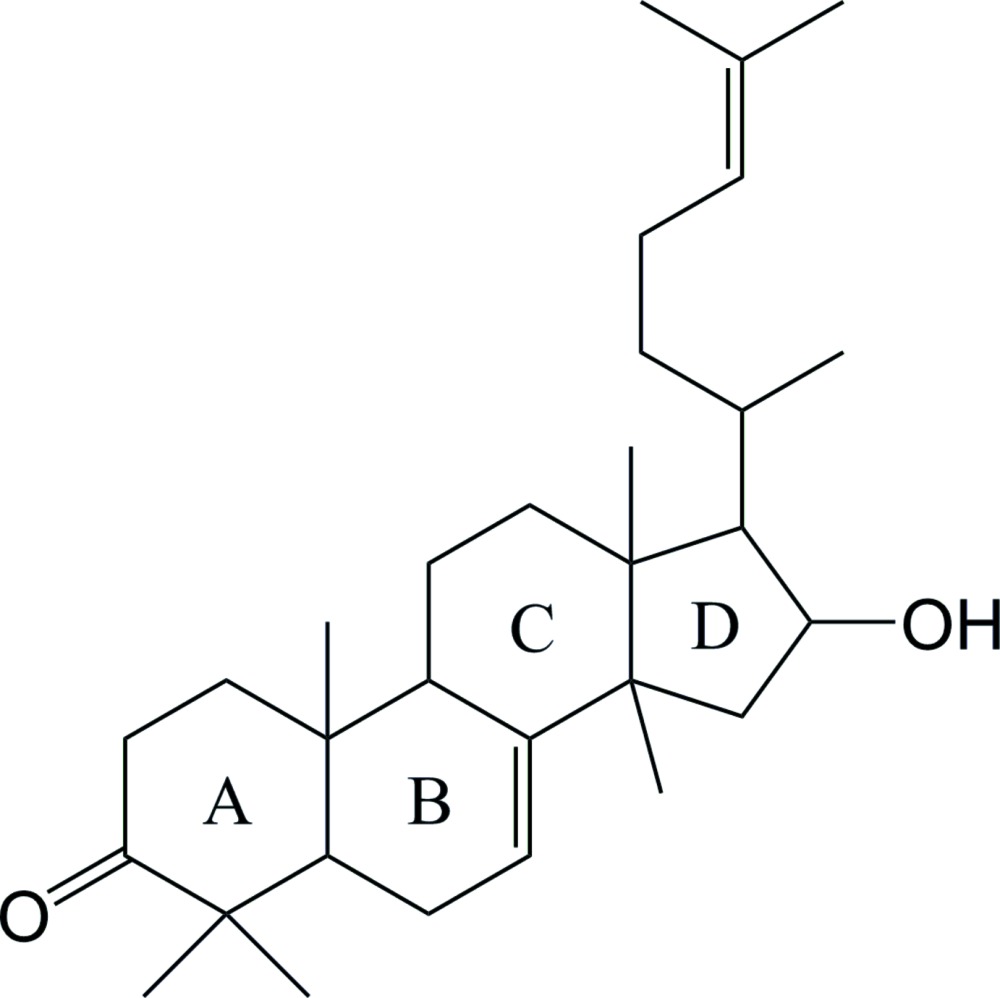


## Experimental   

### Crystal data  

C_30_H_48_O_2_
*M* *_r_* = 440.68
Orthorhombic, 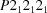

*a* = 12.436 (7) Å
*b* = 13.571 (7) Å
*c* = 16.159 (9) Å
*V* = 2727 (3) Å^3^
*Z* = 4
Mo *K*α radiation
μ = 0.07 mm^−1^
*T* = 298 K
0.31 × 0.25 × 0.22 mm

### Data collection  

Bruker APEXII area-detector diffractometer
Absorption correction: multi-scan (*SADABS*; Bruker, 2002 ▸) *T*_min_ = 0.980, *T*_max_ = 0.986
12873 measured reflections
3000 independent reflections
2320 reflections with *I* > 2σ(*I*)
*R*_int_ = 0.044

### Refinement  

*R*[*F* ^2^ > 2σ(*F* ^2^)] = 0.058
*wR*(*F* ^2^) = 0.198
*S* = 1.08
3000 reflections
298 parameters
H-atom parameters constrained
Δρ_max_ = 0.38 e Å^−3^
Δρ_min_ = −0.30 e Å^−3^

### 

Data collection: *APEX2* (Bruker, 2002 ▸); cell refinement: *SAINT* (Bruker, 2002 ▸); data reduction: *SAINT*; program(s) used to solve structure: *SHELXS97* (Sheldrick, 2008 ▸); program(s) used to refine structure: *SHELXL97* (Sheldrick, 2008 ▸); molecular graphics: *XP* in *SHELXTL* (Sheldrick, 2008 ▸); software used to prepare material for publication: *SHELXL97*.

## Supplementary Material

Crystal structure: contains datablock(s) I, global. DOI: 10.1107/S2056989015010592/xu5852sup1.cif

Structure factors: contains datablock(s) I. DOI: 10.1107/S2056989015010592/xu5852Isup2.hkl

**Fig. 1.**
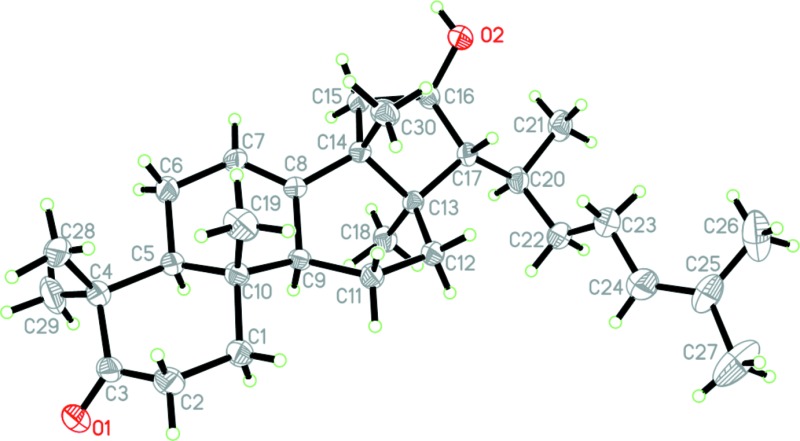
The molecular structure of (I) with the atom numbering, showing displacement ellipsoids at the 50% probability level.

. DOI: 10.1107/S2056989015010592/xu5852fig1.tif
The mol­ecular structure of (I) with the atom numbering, showing displacement ellipsoids at the 50% probability level.

CCDC reference: 1404443

Additional supporting information:  crystallographic information; 3D view; checkCIF report

## Figures and Tables

**Table 1:** Hydrogen-bond geometry (, )

*D*H*A*	*D*H	H*A*	*D* *A*	*D*H*A*
O2H2O1^i^	0.82	2.11	2.894(4)	160

Symmetry code: (i) 
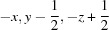
.

## supplementary crystallographic information

### S1. Introduction

Melia azedarach Linn. (Meliaceae), a high tree, enjoys a broad distribution in the most parts of China. The triterpenoids which isolated from this plant is well known for its pharmacological properties, such as analgesic, anti­cancer, anti­viral, anti­malarial, anti­bacterial and anti­feedant activities (Faizi *et al.*, 2002; Wang *et al.*, 2011; Dong *et al.*, 2012). The title compound, 16-hy­droxy-4,4,10,13,14-penta­methyl -17-(6-methyl­hept-5-en-2-yl)-4,5,6,9,10,11,12,13,14,15,16,17-dodeca­hydro -1H-cyclo­penta [*α*]phenanthren-3(2H)-one (I) (Fig. 1) was isolated from the barks of Melia azedarach (Chiang & Chang, 1969). In this work, we obtained a single-crystal of (I) and present here its crystal structure.

The title compound contains a fused four-ring triterpenoid system. rings A adopt a chair conformation, while ring B with one double bond adopts a half-chair conformation, ring C adopts a twist boat conformation and ring D adopts an envelope conformation. Inter­molecular O—H···O hydrogen bonds are present in the crystal structure (Table 1).

### S2. Isolation and crystallization

The air-dried and powered barks of Melia azedarach L.(10.6kg) were percolated with 95% aqueous ethanol for 7 days at room temperature for three times. After evaporation of the solvent under reduced pressure, the gummy residue was suspended in water and then partitioned with EtOAc. The EtOAc extract (145g) was subjected to CC on silica gel eluting with petroleum ether-EtOAc (from 20:1 to 2:1, v/v) to give fifteen fractions (1-15). Fraction 5 (22g) was further separated on silica gel CC and eluted with petroleum-acetone from 20:1 to 3:1, yielding five sub-fractions (2a-2e). Sub-fraction 2c (3.25g), subjected to a series of purification steps using silica gel CC, Sephadex LH-20 to afford Sub-fraction 2c-b-b-b (120.4mg) , then use semi-preparative HPLC (MeCN/H2O 90:10, flow rate 3.8 mL/min) to afford (I) (16.8mg, t _R_ = 32.5min). The structures of (I) was elucidated by means of NMR. Colourless crystal were obtained in the freezer after one month by slow evaporation from acetone/methanol [10:1 (v/v)] mixture solution.

### S3. Refinement

H-atoms bound to carbon were placed in calculated positions with C—H = 0.93–0.98 Å, and refined in riding mode with U_iso_(H) = 1.5U_eq_(C) for methyl H atoms and 1.2U_eq_(C) for the others. Hy­droxy H atoms was placed in calculated position with O—-H = 0.82 Å, and refined in riding mode with U_iso_(H) = 1.5U_eq_(O). The absolute structure has not been determined as no significant anomalous scattering, equivalent diffractions were merged.

### Crystal data

**Table:**

C_30_H_48_O_2_	*F*(000) = 976
*M**_r_* = 440.68	*D*_x_ = 1.073 Mg m^−^^3^
Orthorhombic, *P*2_1_2_1_2_1_	Mo *K*α radiation, λ = 0.71073 Å
Hall symbol: P 2ac 2ab	Cell parameters from 2467 reflections
*a* = 12.436 (7) Å	θ = 2.5–21.6°
*b* = 13.571 (7) Å	µ = 0.07 mm^−^^1^
*c* = 16.159 (9) Å	*T* = 298 K
*V* = 2727 (3) Å^3^	Block, colorless
*Z* = 4	0.31 × 0.25 × 0.22 mm

### Data collection

**Table:**

Bruker APEXII area-detector diffractometer	3000 independent reflections
Radiation source: fine-focus sealed tube	2320 reflections with *I* > 2σ(*I*)
Graphite monochromator	*R*_int_ = 0.044
φ and ω scans	θ_max_ = 26.0°, θ_min_ = 2.0°
Absorption correction: multi-scan (*SADABS*; Bruker, 2002)	*h* = −15→13
*T*_min_ = 0.980, *T*_max_ = 0.986	*k* = −16→16
12873 measured reflections	*l* = −18→19

### Refinement

**Table:**

Refinement on *F*^2^	Primary atom site location: structure-invariant direct methods
Least-squares matrix: full	Secondary atom site location: difference Fourier map
*R*[*F*^2^ > 2σ(*F*^2^)] = 0.058	Hydrogen site location: inferred from neighbouring sites
*wR*(*F*^2^) = 0.198	H-atom parameters constrained
*S* = 1.08	*w* = 1/[σ^2^(*F*_o_^2^) + (0.1351*P*)^2^ + 0.062*P*] where *P* = (*F*_o_^2^ + 2*F*_c_^2^)/3
3000 reflections	(Δ/σ)_max_ < 0.001
298 parameters	Δρ_max_ = 0.38 e Å^−^^3^
0 restraints	Δρ_min_ = −0.30 e Å^−^^3^

### Special details

**Table:**

Geometry. All e.s.d.'s (except the e.s.d. in the dihedral angle between two l.s. planes) are estimated using the full covariance matrix. The cell e.s.d.'s are taken into account individually in the estimation of e.s.d.'s in distances, angles and torsion angles; correlations between e.s.d.'s in cell parameters are only used when they are defined by crystal symmetry. An approximate (isotropic) treatment of cell e.s.d.'s is used for estimating e.s.d.'s involving l.s. planes.
Refinement. Refinement of *F*^2^ against ALL reflections. The weighted *R*-factor *wR* and goodness of fit *S* are based on *F*^2^, conventional *R*-factors *R* are based on *F*, with *F* set to zero for negative *F*^2^. The threshold expression of *F*^2^ > σ(*F*^2^) is used only for calculating *R*-factors(gt) *etc*. and is not relevant to the choice of reflections for refinement. *R*-factors based on *F*^2^ are statistically about twice as large as those based on *F*, and *R*-factors based on ALL data will be even larger.

### Fractional atomic coordinates and isotropic or equivalent isotropic displacement parameters (Å^2^)

**Table:**

	*x*	*y*	*z*	*U*_iso_*/*U*_eq_	
O1	−0.2176 (3)	1.0719 (2)	0.49312 (19)	0.0701 (10)	
O2	0.2062 (3)	0.7741 (2)	−0.04929 (17)	0.0596 (8)	
H2	0.2257	0.7200	−0.0324	0.089*	
C1	−0.2070 (3)	1.0561 (3)	0.2829 (2)	0.0491 (10)	
H1A	−0.1553	1.1080	0.2938	0.059*	
H1B	−0.2475	1.0746	0.2341	0.059*	
C2	−0.2841 (4)	1.0484 (3)	0.3565 (3)	0.0603 (12)	
H2A	−0.3195	1.1112	0.3656	0.072*	
H2B	−0.3389	0.9993	0.3452	0.072*	
C3	−0.2204 (3)	1.0194 (3)	0.4330 (3)	0.0496 (10)	
C4	−0.1582 (3)	0.9224 (3)	0.4286 (3)	0.0507 (10)	
C5	−0.0913 (3)	0.9245 (3)	0.3454 (2)	0.0403 (8)	
H5	−0.0350	0.9736	0.3554	0.048*	
C6	−0.0298 (4)	0.8278 (3)	0.3325 (3)	0.0593 (12)	
H6A	0.0226	0.8201	0.3766	0.071*	
H6B	−0.0798	0.7731	0.3361	0.071*	
C7	0.0274 (3)	0.8238 (3)	0.2502 (3)	0.0498 (10)	
H7	0.0736	0.7710	0.2410	0.060*	
C8	0.0173 (3)	0.8893 (2)	0.1897 (2)	0.0353 (7)	
C9	−0.0524 (3)	0.9818 (2)	0.2036 (2)	0.0332 (7)	
H9	−0.0059	1.0295	0.2318	0.040*	
C10	−0.1457 (3)	0.9588 (2)	0.2652 (2)	0.0383 (8)	
C11	−0.0870 (3)	1.0312 (3)	0.1222 (2)	0.0445 (9)	
H11A	−0.1148	1.0961	0.1352	0.053*	
H11B	−0.1458	0.9932	0.0990	0.053*	
C12	0.0017 (3)	1.0428 (3)	0.0540 (2)	0.0440 (9)	
H12A	−0.0299	1.0277	0.0006	0.053*	
H12B	0.0249	1.1110	0.0527	0.053*	
C13	0.1008 (3)	0.9767 (2)	0.0667 (2)	0.0343 (7)	
C14	0.0645 (3)	0.8748 (2)	0.1044 (2)	0.0346 (7)	
C15	0.1679 (3)	0.8139 (3)	0.0979 (2)	0.0435 (9)	
H15A	0.1517	0.7440	0.0976	0.052*	
H15B	0.2157	0.8280	0.1438	0.052*	
C16	0.2196 (3)	0.8456 (3)	0.0147 (2)	0.0436 (9)	
H16	0.2964	0.8585	0.0231	0.052*	
C17	0.1624 (3)	0.9421 (2)	−0.0125 (2)	0.0362 (7)	
H17	0.1076	0.9229	−0.0530	0.043*	
C18	0.1798 (3)	1.0301 (3)	0.1263 (2)	0.0454 (9)	
H18A	0.2058	1.0892	0.1005	0.068*	
H18B	0.2393	0.9874	0.1384	0.068*	
H18C	0.1431	1.0465	0.1767	0.068*	
C19	−0.2237 (4)	0.8835 (3)	0.2255 (3)	0.0625 (12)	
H19A	−0.1870	0.8221	0.2171	0.094*	
H19B	−0.2485	0.9084	0.1732	0.094*	
H19C	−0.2841	0.8733	0.2615	0.094*	
C20	0.2391 (3)	1.0155 (3)	−0.0574 (2)	0.0470 (9)	
H20	0.2945	1.0353	−0.0176	0.056*	
C21	0.2962 (4)	0.9630 (4)	−0.1298 (3)	0.0590 (11)	
H21A	0.2434	0.9361	−0.1668	0.089*	
H21B	0.3403	0.9107	−0.1086	0.089*	
H21C	0.3404	1.0094	−0.1590	0.089*	
C22	0.1849 (4)	1.1094 (3)	−0.0890 (3)	0.0569 (11)	
H22A	0.1508	1.1419	−0.0424	0.068*	
H22B	0.2403	1.1532	−0.1096	0.068*	
C23	0.1024 (5)	1.0964 (3)	−0.1554 (3)	0.0704 (14)	
H23A	0.0522	1.0455	−0.1385	0.085*	
H23B	0.1380	1.0739	−0.2054	0.085*	
C24	0.0405 (6)	1.1890 (4)	−0.1748 (4)	0.0844 (17)	
H24	0.0212	1.2267	−0.1290	0.101*	
C25	0.0095 (4)	1.2247 (4)	−0.2468 (4)	0.0774 (15)	
C26	0.0271 (5)	1.1679 (7)	−0.3249 (4)	0.111 (3)	
H26A	0.0672	1.1092	−0.3129	0.166*	
H26B	0.0666	1.2077	−0.3635	0.166*	
H26C	−0.0411	1.1504	−0.3485	0.166*	
C27	−0.0482 (6)	1.3212 (5)	−0.2542 (6)	0.129 (3)	
H27A	−0.0586	1.3488	−0.2001	0.193*	
H27B	−0.1168	1.3109	−0.2801	0.193*	
H27C	−0.0061	1.3657	−0.2872	0.193*	
C28	−0.2423 (5)	0.8383 (3)	0.4348 (3)	0.0740 (15)	
H28A	−0.2846	0.8469	0.4840	0.111*	
H28B	−0.2060	0.7760	0.4371	0.111*	
H28C	−0.2885	0.8400	0.3872	0.111*	
C29	−0.0838 (5)	0.9145 (5)	0.5010 (3)	0.0841 (17)	
H29A	−0.0379	0.9713	0.5028	0.126*	
H29B	−0.0406	0.8562	0.4955	0.126*	
H29C	−0.1250	0.9108	0.5511	0.126*	
C30	−0.0204 (3)	0.8237 (3)	0.0497 (3)	0.0493 (9)	
H30A	−0.0429	0.7635	0.0756	0.074*	
H30B	0.0102	0.8094	−0.0035	0.074*	
H30C	−0.0813	0.8664	0.0430	0.074*	

### Atomic displacement parameters (Å^2^)

**Table:**

	*U*^11^	*U*^22^	*U*^33^	*U*^12^	*U*^13^	*U*^23^
O1	0.106 (3)	0.0522 (16)	0.0525 (18)	0.0022 (17)	0.0278 (19)	−0.0093 (15)
O2	0.088 (2)	0.0471 (14)	0.0433 (15)	0.0172 (15)	0.0117 (15)	−0.0010 (12)
C1	0.049 (2)	0.051 (2)	0.048 (2)	0.0132 (18)	0.0126 (18)	0.0032 (18)
C2	0.056 (2)	0.057 (2)	0.069 (3)	0.016 (2)	0.023 (2)	0.003 (2)
C3	0.057 (2)	0.0394 (18)	0.052 (2)	−0.0032 (17)	0.0254 (19)	−0.0021 (18)
C4	0.064 (2)	0.0424 (19)	0.046 (2)	0.0032 (18)	0.018 (2)	0.0011 (17)
C5	0.0451 (19)	0.0362 (17)	0.0396 (19)	−0.0001 (16)	0.0070 (16)	−0.0007 (15)
C6	0.079 (3)	0.052 (2)	0.048 (2)	0.025 (2)	0.023 (2)	0.0116 (19)
C7	0.062 (2)	0.0406 (18)	0.046 (2)	0.0200 (18)	0.016 (2)	0.0088 (17)
C8	0.0376 (17)	0.0309 (15)	0.0373 (18)	0.0029 (13)	0.0015 (15)	−0.0008 (14)
C9	0.0349 (16)	0.0326 (15)	0.0322 (17)	0.0033 (13)	0.0004 (14)	−0.0018 (14)
C10	0.0373 (17)	0.0372 (17)	0.0403 (19)	0.0001 (14)	0.0029 (15)	−0.0025 (15)
C11	0.049 (2)	0.048 (2)	0.0363 (19)	0.0168 (17)	−0.0024 (16)	0.0032 (16)
C12	0.051 (2)	0.0444 (18)	0.0362 (18)	0.0123 (16)	−0.0010 (17)	0.0016 (16)
C13	0.0387 (16)	0.0327 (15)	0.0316 (17)	−0.0019 (13)	−0.0055 (14)	0.0021 (14)
C14	0.0376 (17)	0.0313 (15)	0.0348 (18)	0.0023 (13)	0.0033 (15)	0.0006 (14)
C15	0.054 (2)	0.0378 (17)	0.039 (2)	0.0103 (16)	0.0049 (17)	0.0046 (15)
C16	0.046 (2)	0.0493 (19)	0.0354 (19)	0.0110 (16)	0.0054 (16)	0.0016 (16)
C17	0.0380 (17)	0.0413 (17)	0.0292 (16)	0.0009 (14)	−0.0023 (14)	0.0015 (14)
C18	0.046 (2)	0.050 (2)	0.040 (2)	−0.0088 (17)	−0.0043 (16)	−0.0036 (17)
C19	0.051 (2)	0.067 (3)	0.069 (3)	−0.020 (2)	0.009 (2)	−0.015 (2)
C20	0.052 (2)	0.0485 (19)	0.040 (2)	−0.0078 (17)	0.0036 (17)	0.0050 (17)
C21	0.054 (2)	0.073 (3)	0.050 (2)	0.004 (2)	0.017 (2)	0.014 (2)
C22	0.074 (3)	0.045 (2)	0.052 (2)	−0.0038 (19)	0.010 (2)	0.0076 (19)
C23	0.103 (4)	0.056 (2)	0.053 (3)	0.004 (3)	−0.001 (3)	0.005 (2)
C24	0.114 (4)	0.069 (3)	0.071 (3)	0.019 (3)	−0.017 (3)	0.000 (3)
C25	0.063 (3)	0.082 (3)	0.087 (4)	0.008 (3)	−0.011 (3)	0.034 (3)
C26	0.085 (4)	0.187 (8)	0.060 (3)	0.028 (5)	0.001 (3)	0.027 (4)
C27	0.099 (5)	0.088 (4)	0.199 (8)	0.003 (4)	−0.048 (6)	0.060 (5)
C28	0.103 (4)	0.045 (2)	0.074 (3)	−0.007 (2)	0.044 (3)	0.007 (2)
C29	0.101 (4)	0.115 (4)	0.036 (2)	0.026 (4)	0.017 (3)	0.006 (3)
C30	0.052 (2)	0.0454 (19)	0.050 (2)	−0.0099 (17)	0.0067 (19)	−0.0096 (18)

### Geometric parameters (Å, º)

**Table:**

O1—C3	1.206 (5)	C16—C17	1.553 (5)
O2—C16	1.428 (4)	C16—H16	0.9800
O2—H2	0.8200	C17—C20	1.558 (5)
C1—C2	1.531 (5)	C17—H17	0.9800
C1—C10	1.552 (5)	C18—H18A	0.9600
C1—H1A	0.9700	C18—H18B	0.9600
C1—H1B	0.9700	C18—H18C	0.9600
C2—C3	1.521 (6)	C19—H19A	0.9600
C2—H2A	0.9700	C19—H19B	0.9600
C2—H2B	0.9700	C19—H19C	0.9600
C3—C4	1.528 (5)	C20—C22	1.529 (6)
C4—C29	1.495 (7)	C20—C21	1.543 (6)
C4—C28	1.552 (6)	C20—H20	0.9800
C4—C5	1.581 (5)	C21—H21A	0.9600
C5—C6	1.533 (5)	C21—H21B	0.9600
C5—C10	1.535 (5)	C21—H21C	0.9600
C5—H5	0.9800	C22—C23	1.495 (7)
C6—C7	1.510 (6)	C22—H22A	0.9700
C6—H6A	0.9700	C22—H22B	0.9700
C6—H6B	0.9700	C23—C24	1.507 (7)
C7—C8	1.327 (5)	C23—H23A	0.9700
C7—H7	0.9300	C23—H23B	0.9700
C8—C14	1.510 (5)	C24—C25	1.318 (8)
C8—C9	1.541 (4)	C24—H24	0.9300
C9—C11	1.537 (5)	C25—C26	1.495 (10)
C9—C10	1.560 (5)	C25—C27	1.498 (8)
C9—H9	0.9800	C26—H26A	0.9600
C10—C19	1.549 (5)	C26—H26B	0.9600
C11—C12	1.568 (5)	C26—H26C	0.9600
C11—H11A	0.9700	C27—H27A	0.9600
C11—H11B	0.9700	C27—H27B	0.9600
C12—C13	1.537 (5)	C27—H27C	0.9600
C12—H12A	0.9700	C28—H28A	0.9600
C12—H12B	0.9700	C28—H28B	0.9600
C13—C18	1.554 (5)	C28—H28C	0.9600
C13—C17	1.564 (5)	C29—H29A	0.9600
C13—C14	1.577 (5)	C29—H29B	0.9600
C14—C15	1.532 (5)	C29—H29C	0.9600
C14—C30	1.542 (5)	C30—H30A	0.9600
C15—C16	1.550 (5)	C30—H30B	0.9600
C15—H15A	0.9700	C30—H30C	0.9600
C15—H15B	0.9700		
			
C16—O2—H2	109.5	O2—C16—C17	108.3 (3)
C2—C1—C10	113.1 (3)	C15—C16—C17	106.8 (3)
C2—C1—H1A	108.9	O2—C16—H16	109.5
C10—C1—H1A	109.0	C15—C16—H16	109.5
C2—C1—H1B	109.0	C17—C16—H16	109.5
C10—C1—H1B	108.9	C16—C17—C20	113.0 (3)
H1A—C1—H1B	107.8	C16—C17—C13	104.2 (3)
C3—C2—C1	108.8 (3)	C20—C17—C13	119.2 (3)
C3—C2—H2A	109.9	C16—C17—H17	106.5
C1—C2—H2A	109.9	C20—C17—H17	106.5
C3—C2—H2B	109.9	C13—C17—H17	106.5
C1—C2—H2B	109.9	C13—C18—H18A	109.5
H2A—C2—H2B	108.3	C13—C18—H18B	109.5
O1—C3—C2	121.1 (4)	H18A—C18—H18B	109.5
O1—C3—C4	122.2 (4)	C13—C18—H18C	109.5
C2—C3—C4	116.7 (3)	H18A—C18—H18C	109.5
C29—C4—C3	109.8 (4)	H18B—C18—H18C	109.5
C29—C4—C28	108.3 (4)	C10—C19—H19A	109.5
C3—C4—C28	106.9 (3)	C10—C19—H19B	109.5
C29—C4—C5	109.9 (3)	H19A—C19—H19B	109.5
C3—C4—C5	106.9 (3)	C10—C19—H19C	109.5
C28—C4—C5	115.0 (3)	H19A—C19—H19C	109.5
C6—C5—C10	111.4 (3)	H19B—C19—H19C	109.5
C6—C5—C4	111.3 (3)	C22—C20—C21	109.5 (3)
C10—C5—C4	119.4 (3)	C22—C20—C17	114.7 (3)
C6—C5—H5	104.3	C21—C20—C17	109.8 (3)
C10—C5—H5	104.3	C22—C20—H20	107.5
C4—C5—H5	104.3	C21—C20—H20	107.5
C7—C6—C5	112.7 (3)	C17—C20—H20	107.5
C7—C6—H6A	109.1	C20—C21—H21A	109.5
C5—C6—H6A	109.1	C20—C21—H21B	109.5
C7—C6—H6B	109.1	H21A—C21—H21B	109.5
C5—C6—H6B	109.1	C20—C21—H21C	109.5
H6A—C6—H6B	107.8	H21A—C21—H21C	109.5
C8—C7—C6	125.5 (3)	H21B—C21—H21C	109.5
C8—C7—H7	117.2	C23—C22—C20	116.4 (4)
C6—C7—H7	117.2	C23—C22—H22A	108.2
C7—C8—C14	123.2 (3)	C20—C22—H22A	108.2
C7—C8—C9	119.4 (3)	C23—C22—H22B	108.2
C14—C8—C9	117.2 (3)	C20—C22—H22B	108.2
C11—C9—C8	112.8 (3)	H22A—C22—H22B	107.3
C11—C9—C10	115.1 (3)	C22—C23—C24	113.7 (4)
C8—C9—C10	110.4 (3)	C22—C23—H23A	108.8
C11—C9—H9	105.9	C24—C23—H23A	108.8
C8—C9—H9	105.9	C22—C23—H23B	108.8
C10—C9—H9	105.9	C24—C23—H23B	108.8
C5—C10—C19	115.2 (3)	H23A—C23—H23B	107.7
C5—C10—C1	108.6 (3)	C25—C24—C23	129.7 (6)
C19—C10—C1	109.3 (3)	C25—C24—H24	115.1
C5—C10—C9	105.8 (3)	C23—C24—H24	115.1
C19—C10—C9	109.5 (3)	C24—C25—C26	120.9 (5)
C1—C10—C9	108.3 (3)	C24—C25—C27	122.1 (6)
C9—C11—C12	116.6 (3)	C26—C25—C27	116.9 (6)
C9—C11—H11A	108.1	C25—C26—H26A	109.5
C12—C11—H11A	108.1	C25—C26—H26B	109.5
C9—C11—H11B	108.1	H26A—C26—H26B	109.5
C12—C11—H11B	108.1	C25—C26—H26C	109.5
H11A—C11—H11B	107.3	H26A—C26—H26C	109.5
C13—C12—C11	114.3 (3)	H26B—C26—H26C	109.5
C13—C12—H12A	108.7	C25—C27—H27A	109.5
C11—C12—H12A	108.7	C25—C27—H27B	109.5
C13—C12—H12B	108.7	H27A—C27—H27B	109.5
C11—C12—H12B	108.7	C25—C27—H27C	109.5
H12A—C12—H12B	107.6	H27A—C27—H27C	109.5
C12—C13—C18	108.6 (3)	H27B—C27—H27C	109.5
C12—C13—C17	117.3 (3)	C4—C28—H28A	109.5
C18—C13—C17	109.7 (3)	C4—C28—H28B	109.5
C12—C13—C14	109.5 (3)	H28A—C28—H28B	109.5
C18—C13—C14	110.4 (3)	C4—C28—H28C	109.5
C17—C13—C14	101.1 (2)	H28A—C28—H28C	109.5
C8—C14—C15	117.4 (3)	H28B—C28—H28C	109.5
C8—C14—C30	108.4 (3)	C4—C29—H29A	109.5
C15—C14—C30	107.0 (3)	C4—C29—H29B	109.5
C8—C14—C13	110.5 (3)	H29A—C29—H29B	109.5
C15—C14—C13	101.9 (3)	C4—C29—H29C	109.5
C30—C14—C13	111.6 (3)	H29A—C29—H29C	109.5
C14—C15—C16	105.0 (3)	H29B—C29—H29C	109.5
C14—C15—H15A	110.8	C14—C30—H30A	109.5
C16—C15—H15A	110.8	C14—C30—H30B	109.5
C14—C15—H15B	110.8	H30A—C30—H30B	109.5
C16—C15—H15B	110.8	C14—C30—H30C	109.5
H15A—C15—H15B	108.8	H30A—C30—H30C	109.5
O2—C16—C15	113.0 (3)	H30B—C30—H30C	109.5
			
C10—C1—C2—C3	58.6 (5)	C11—C12—C13—C17	150.0 (3)
C1—C2—C3—O1	119.7 (4)	C11—C12—C13—C14	35.6 (4)
C1—C2—C3—C4	−58.8 (5)	C7—C8—C14—C15	−30.3 (5)
O1—C3—C4—C29	−9.4 (5)	C9—C8—C14—C15	154.2 (3)
C2—C3—C4—C29	169.0 (4)	C7—C8—C14—C30	90.9 (4)
O1—C3—C4—C28	107.8 (5)	C9—C8—C14—C30	−84.6 (4)
C2—C3—C4—C28	−73.8 (4)	C7—C8—C14—C13	−146.5 (4)
O1—C3—C4—C5	−128.6 (4)	C9—C8—C14—C13	38.0 (4)
C2—C3—C4—C5	49.8 (4)	C12—C13—C14—C8	−64.4 (3)
C29—C4—C5—C6	63.7 (5)	C18—C13—C14—C8	55.1 (4)
C3—C4—C5—C6	−177.2 (4)	C17—C13—C14—C8	171.2 (3)
C28—C4—C5—C6	−58.7 (5)	C12—C13—C14—C15	170.2 (3)
C29—C4—C5—C10	−164.2 (4)	C18—C13—C14—C15	−70.3 (3)
C3—C4—C5—C10	−45.1 (4)	C17—C13—C14—C15	45.7 (3)
C28—C4—C5—C10	73.3 (4)	C12—C13—C14—C30	56.3 (4)
C10—C5—C6—C7	39.1 (5)	C18—C13—C14—C30	175.8 (3)
C4—C5—C6—C7	175.1 (4)	C17—C13—C14—C30	−68.1 (3)
C5—C6—C7—C8	−7.9 (7)	C8—C14—C15—C16	−158.2 (3)
C6—C7—C8—C14	−171.4 (4)	C30—C14—C15—C16	79.8 (3)
C6—C7—C8—C9	3.9 (7)	C13—C14—C15—C16	−37.4 (3)
C7—C8—C9—C11	−160.7 (4)	C14—C15—C16—O2	−104.2 (3)
C14—C8—C9—C11	15.0 (4)	C14—C15—C16—C17	14.8 (4)
C7—C8—C9—C10	−30.4 (5)	O2—C16—C17—C20	−93.0 (4)
C14—C8—C9—C10	145.3 (3)	C15—C16—C17—C20	145.0 (3)
C6—C5—C10—C19	56.6 (4)	O2—C16—C17—C13	136.1 (3)
C4—C5—C10—C19	−75.4 (4)	C15—C16—C17—C13	14.1 (4)
C6—C5—C10—C1	179.5 (3)	C12—C13—C17—C16	−155.4 (3)
C4—C5—C10—C1	47.5 (4)	C18—C13—C17—C16	80.2 (3)
C6—C5—C10—C9	−64.5 (4)	C14—C13—C17—C16	−36.4 (3)
C4—C5—C10—C9	163.5 (3)	C12—C13—C17—C20	77.4 (4)
C2—C1—C10—C5	−52.9 (4)	C18—C13—C17—C20	−47.0 (4)
C2—C1—C10—C19	73.6 (5)	C14—C13—C17—C20	−163.6 (3)
C2—C1—C10—C9	−167.3 (3)	C16—C17—C20—C22	177.2 (3)
C11—C9—C10—C5	−172.0 (3)	C13—C17—C20—C22	−59.9 (4)
C8—C9—C10—C5	58.8 (3)	C16—C17—C20—C21	53.3 (4)
C11—C9—C10—C19	63.3 (4)	C13—C17—C20—C21	176.3 (3)
C8—C9—C10—C19	−65.8 (4)	C21—C20—C22—C23	59.2 (5)
C11—C9—C10—C1	−55.8 (4)	C17—C20—C22—C23	−64.8 (5)
C8—C9—C10—C1	175.1 (3)	C20—C22—C23—C24	171.4 (4)
C8—C9—C11—C12	−44.3 (4)	C22—C23—C24—C25	139.4 (7)
C10—C9—C11—C12	−172.2 (3)	C23—C24—C25—C26	5.0 (11)
C9—C11—C12—C13	17.4 (5)	C23—C24—C25—C27	−177.4 (6)
C11—C12—C13—C18	−85.0 (4)		

### Hydrogen-bond geometry (Å, º)

**Table:**

*D*—H···*A*	*D*—H	H···*A*	*D*···*A*	*D*—H···*A*
O2—H2···O1^i^	0.82	2.11	2.894 (4)	160

Symmetry code: (i) −*x*, *y*−1/2, −*z*+1/2.
