# Supplementary figures and images for: Crystal structure of 16-hy­droxy-4,4,10,13,14-penta­methyl-17-(6-methyl­hept-5-en-2-yl)-4,5,6,9,10,11,12,13,14,15,16,17-dodeca­hydro-1H-cyclo­penta­[a]phenanthren-3(2H)-one
Source: Acta Crystallogr E Crystallogr Commun. 2015 Jun 13;71(Pt 7):o464–5. doi: 10.1107/S2056989015010592 (PMC4518949; doi:10.1107/S2056989015010592)

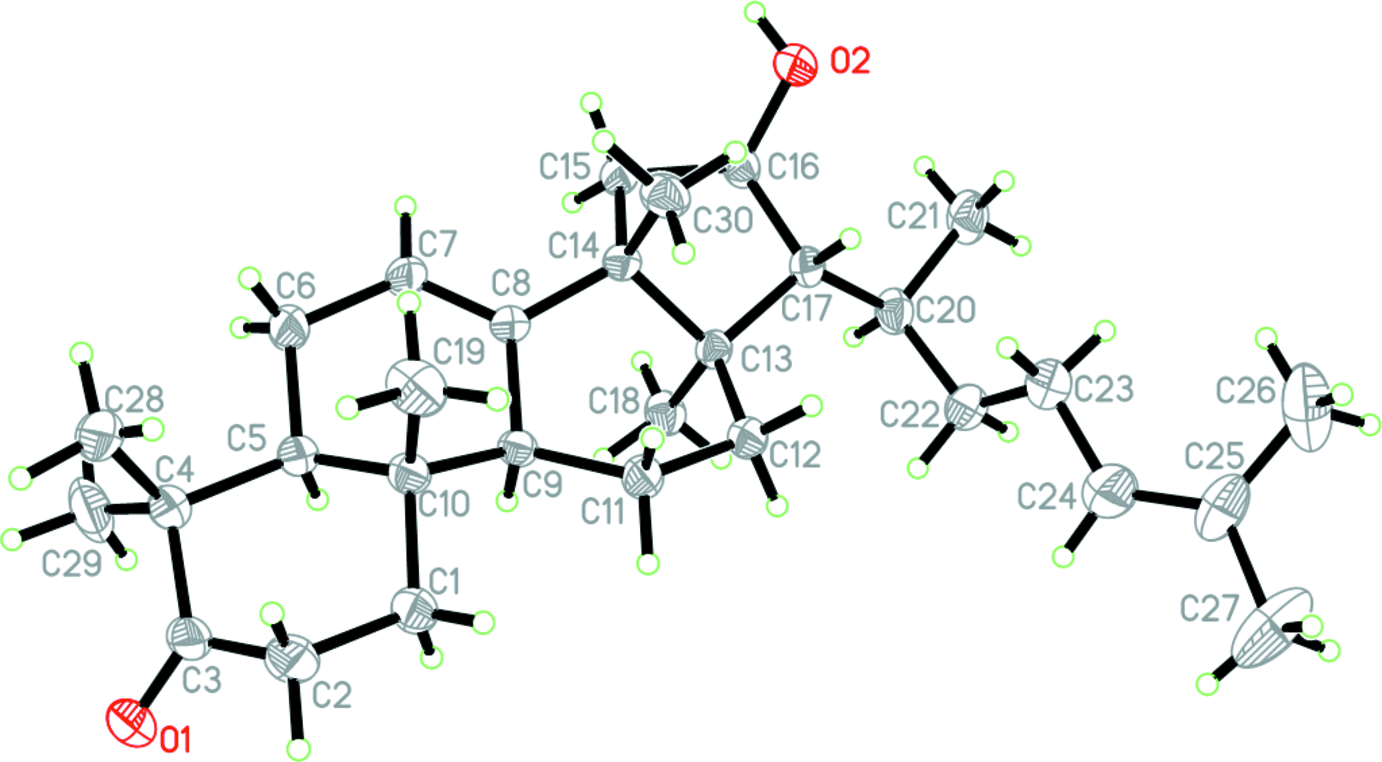

Supplement: Supplementary file 3 [file e-71-0o464-fig1.tif]
